# Autologous Versus Implant-Based Breast Reconstruction: Insights From Recent Oncologic and Plastic Surgery Guidelines

**DOI:** 10.7759/cureus.109474

**Published:** 2026-05-23

**Authors:** Esaúl Marroquín León, Daniel Sanchez, Ángel Gutiérrez Lamont, Sofía Rodríguez Palomino, Diana Laura Molina Medina, Olimpia Alejandra Méndez Campos, Marion Camacho Escárcega, Mariana Alcalá, Alexa Herrera Cohen, Xavier Antonio Sanchez Garcia

**Affiliations:** 1 Medicine, Universidad Westhill, Mexico City, MEX; 2 Research, Escuela Superior de Medicina, Instituto Politécnico Nacional, Mexico City, MEX; 3 Research, Universidad Anáhuac México Norte, Mexico City, MEX; 4 Research, Centro de Estudios Superiores de Tepeaca, Puebla, MEX; 5 Research, Universidad La Salle, Mexico City, MEX; 6 Research, Universidad Westhill, Mexico City, MEX; 7 Plastic and Reconstructive Surgery, Hospital Ángeles Pedregal, Mexico City, MEX; 8 Plastic and Reconstructive Surgery, Hospital Central Militar, Mexico City, MEX

**Keywords:** adjuvant radiotherapy, autologous transplantation, breast implants, breast reconstruction, clinical practice guidelines, mastectomy, patient reported outcome measures, prepectoral breast reconstruction

## Abstract

Mastectomy rates remain high in the management of breast cancer, which reflects the evolution of post-mastectomy breast reconstruction into a critical component of multidisciplinary care. The two primary methods for breast reconstruction consist of using the patient's own tissue (autologous) and implant-based procedures. The selection between the two procedures depends on three main factors, which include oncological requirements, surgical risk, and patient preferences.

This article aims to synthesize recent clinical guidelines and literature to provide an evidence-based comparison of these surgical modalities. A comprehensive literature search was conducted to find current clinical practice guidelines and high-quality clinical studies about post-mastectomy breast reconstruction. The research assessed how cancer treatments affect patients through their evaluation of radiotherapy effects and complication rates, and patient-reported outcome measures (PROMs), which used the BREAST-Q tool for both autologous and implant-based groups. The use of prepectoral implants decreased pain and animation deformity but showed higher seroma rates and can also fail with radiotherapy. Autologous reconstruction provides superior long-term psychosocial and sexual life quality. The two main factors that determine reconstructive failure success rates include patients who are obese and smokers.

## Introduction and background

Breast cancer is a malignant neoplasia characterized by the uncontrolled growth of cells in mammary tissue. According to World Health Organization (WHO) reports, it is known to rank as the most common cancer among women worldwide, as its incidence rises progressively with age and, in general, it is more frequent during menopause, adversely affecting women's health, quality of life, and overall well-being. Research has shown that breast cancer accounts for approximately 33% of cancers diagnosed and 20% of cancer-related deaths in women, which has contributed to an increase in mastectomy rates as part of oncological treatment [1,2].

Currently, there are two principal approaches for post-mastectomy reconstruction: implant-based and autologous breast reconstruction, each with distinct benefits, indications, and limitations. First, implant-based reconstruction is characterized by the use of prosthetic implants. In contrast, autologous reconstruction utilizes the patient's own tissues, e.g., musculocutaneous or perforator flaps, both of which are associated with long-lasting results and a more natural appearance. Both are accepted in current clinical practice and are constantly evolving over time [3-5].

In recent years, organizations such as the American Society for Radiation Oncology (ASTRO), the National Comprehensive Cancer Network® (NCCN®), and the American Society of Plastic Surgeons (ASPS) have addressed key aspects in clinical guidelines regarding post-mastectomy breast reconstruction, principally to emphasize individualized treatment, oncological safety, and optimal reconstruction outcomes. These updates promote a shared decision-making model in which patients’ preferences are respected and not solely based on oncological clinical factors [6-8].

The choice of appropriate approaches for post-mastectomy reconstruction is complex because different methods offer distinct outcomes, although they have specific surgical indications, limitations, and varying safety risks for each patient. This review evaluates how clinical guidelines and research evidence affect clinical decision-making between autologous breast reconstruction and implant-based breast reconstruction through their impact on complication rates and patient satisfaction [9,10].

## Review

Overview of recent guidelines and key recommendations

International oncology boards have revised clinical guidelines on post-mastectomy breast reconstruction in order to underscore a multidisciplinary approach, positioning both autologous and implant-based modalities as tenable clinical pathways, provided they align with patient safety and oncologic and clinical priorities [11]. The NCCN Clinical Practice Guidelines in Oncology (NCCN® Guidelines) posit that reconstruction should not delay or compromise indicated cancer therapy, recommending individualized plans based on patient-specific determinants or risk factors (RFs), such as comorbidities, tobacco use, and elevated body composition metrics, while emphasizing different approaches for immediate or delayed timing [11].

Currently, breast reconstruction has a multidisciplinary approach that focuses on maximum oncological safety while optimizing aesthetic outcomes. In this regard, NCCN highlights immediate or delayed reconstruction as acceptable strategies, with radiation therapy (RT) being a factor in timing and reconstructive approach. Oncoplastic techniques may be considered during breast-conserving surgery (BCS) to optimize cosmetic outcomes. Lastly, if adjuvant RT is required, reconstructive options for addressing subsequent aesthetic or contour defects include delayed fat grafting or delayed flap reconstruction, with the latter typically performed six or more months after completion of RT. Additionally, contralateral reduction or mastopexy may be considered to achieve symmetry [11].

On the other hand, when mastectomy is chosen, decisions are made considering whether adjuvant RT is required, not required, or remains unknown. When adjuvant RT is planned, the reconstruction options include: implant-based reconstruction can be immediate (first stage) or may require the use of an expander before placing the definitive implant (second stage), always avoiding delays in the start of RT due to aesthetic interventions. Meanwhile, when autologous reconstruction is planned, it can be performed immediately, delayed until the end of RT (≥6 months), or, if necessary, a tissue expander can be placed, followed by completion of reconstruction after RT. In cases with a history of RT, either due to recurrent carcinoma or when reconstruction was performed after RT, the soft tissues should be evaluated before and during surgery to determine their suitability; in both cases, autologous reconstruction is preferred, or a combination approach may be used.

When it is unknown whether RT will be necessary, the decision will be based on whether adjuvant chemotherapy is planned or not. In the first case, a tissue expander will be placed, and if RT is not required after completion, the expander will be replaced using either of the two techniques. In the second case, in the absence of RT, the previous indications will be followed. Finally, when inflammatory breast cancer is diagnosed, it is considered a special case, and broadly, the clinical standard is delayed breast reconstruction for various reasons [11]. Table 1 summarizes the key points for clinical practice from the NCCN Guidelines [11].

**Table 1:** NCCN guideline key aspects for clinical practice and treatment options Referenced with permission from the NCCN Clinical Practice Guidelines in Oncology (NCCN Guidelines®) for Breast Cancer, Version 2.2026 [11]. © National Comprehensive Cancer Network, Inc. 2026. All rights reserved. For the most recent and complete version of the guideline, please visit NCCN.org. NCCN makes no warranties of any kind whatsoever regarding their content, use, or application, and disclaims any responsibility for their application or use in any way. RT, Radiotherapy; BCS, Breast-Conserving Surgery; IBC, Inflammatory Breast Cancer; N/A, Not Applicable; LD, Latissimus Dorsi Flap; TDPA, Thoracodorsal Artery Perforator Flap

Clinical Scenario (RT Status)	Soft Tissue Assessment	Primary Reconstruction Strategies	Key Clinical Timing & Pearls
Planned Adjuvant RT	Critical (per-op & intra-op)	Implant-based: 2-stage (Expander → Permanent) or 1-stage (Direct to implant).	Timing: Exchange to permanent implant should occur either prior to RT or ≥6 months after RT completion to minimize complications.
Prior History of RT	Mandatory (adequacy of tissue determines the path)	Autologous: Immediate, delayed (≥6 months post-RT), or expander-to-delayed autologous (≥6 months post-RT). If adequate: autologous (preferred) or combination, 1-stage direct-to-implant (for recurrent carcinoma after breast-conserving therapy including RT), or 2-stage expander-to-implant.	Pearl: RT increases the risk of capsular contracture and reconstructive failure in implant-based cases. Pearl: In patients with prior RT, autologous tissue is often preferred to mitigate the effects of irradiation on prosthetic success.
		If Inadequate: Autologous (preferred) or combination (e.g., Latissimus flap + implant).	Soft Tissue: Irradiated skin has limited elasticity; a flap + tissue expander implant may be required to accommodate a permanent implant.
No / Unknown RT History	Required for 1-stage decisions	Immediate: Tissue expander placement (at time of mastectomy), 1-stage direct to implant, or immediate autologous or LD with implant at the time of mastectomy.	Consider delaying autologous reconstruction until after RT is completed, as RT to a flap may cause loss of cosmesis or fat necrosis.
		Delayed: Options depend on whether RT is eventually required or not.	Logic: If RT is later found to be required, transition to the "Prior History of RT" algorithm. Chemotherapy: If adjuvant chemo is planned, tissue expansion can continue during treatment.
BCS	Pre-operative volume/cosmesis evaluation	Oncoplastic Surgery: Reduction, mastopexy, or local/regional flaps (LD, TDAP).	Requirement: Mark the cavity with clips to assist the radiation oncology team in RT planning.
IBC	N/A	Delayed Reconstruction after mastectomy.	Pearl: High-risk nature of IBC necessitates prioritizing oncologic control before reconstruction.

The NCCN also offers a patient-directed guide, providing key data that should currently be the most important part of treatment: informing the patient about their diagnosis and treatment options to create a physician-patient relationship that benefits both parties and promotes shared and informed decision-making [12].

The ASPS guidelines, for their part, focus mainly on implant-based techniques, advising preoperative education on risks such as obesity and radiation, and suggesting referral to plastic surgery before mastectomy to minimize complications such as infection or capsular contracture [13]. Similarly to the NCCN recommendations, the selection between autologous or implant-based breast reconstruction entirely depends on RT or adjuvant RT prognosis and the relationship between the volume of neoplastic tissue and healthy or remaining tissue. In patients who undergo BCS, oncoplastic techniques are recommended, and, as an additional note, the placement of metal clips is of utmost importance to help identify the surgical site during RT, especially if grafts are used or after radical surgery.

The data show that implant-based reconstruction is the most prevalent option and is preferred in the absence of a history of invasive carcinoma, with outcomes influenced by patient RFs. Complications are greater in smokers (2.2-3 times higher risk than non-smokers), including necrosis, failed reconstruction, or implant rejection. Obesity (BMI > 30) is considered a relative contraindication that increases the risk of implant loss, and the use of acellular dermal matrix (ADM) can optimize outcomes; however, it is associated with a higher incidence of seroma and infection in patients with large breast volume. Similarly, delayed reconstruction is mandatory in inflammatory breast cancer until completion of trimodal oncological treatment, and adjuvant RT should ideally be administered within eight weeks post-mastectomy to avoid compromising recurrence-free survival, regardless of the reconstructive approach [13]. 

Joint guidelines from ASTRO, ASCO, and SSO address post-mastectomy radiation therapy (PMRT). In general, PMRT is recommended for most patients with node-positive breast cancer, and for those with node-negative disease, it will depend on clinical judgment, taking into account the characteristics and needs of each patient. Similarly, neoadjuvant systemic therapy is indicated when the diagnosis includes locally advanced disease and also when there is residual nodal disease at the time of surgery. Furthermore, emphasis is placed on the fact that when PMRT is initiated, treatment of the ipsilateral chest wall or reconstructed breast and regional lymph nodes (axillary, supra-/infraclavicular, or internal mammary nodes) is also indicated. In this case, the preferred treatment is moderate hypofractionation, since oncological outcomes are equivalent and toxicity is lower than with conventional fractionated treatment (2.7% vs 7.7% treatment breaks). It is important to note that none of the reviewed clinical trials addressed cosmetic outcomes and their complications after hypofractionation [14].

Current evidence from systematic reviews supports these recommendations, indicating that autologous flaps may offer better long-term satisfaction and aesthetic results, though at higher initial costs and operative times compared with implants [15]. Both approaches demonstrate similar oncologic safety, with no increased recurrence rates, and complications vary by technique; however, implants predispose patients to higher infection rates, whereas autologous techniques are associated with donor-site morbidity [16]. A meta-analysis that included 32 studies found that autologous reconstruction showed significantly better aesthetic and patient satisfaction outcomes (CI -9.97 to -3.14; p < 0.001) [17]. Similar evidence indicates that autologous methods also perform better in patient-reported outcomes such as psychosocial well-being, while cost-effectiveness favors flaps over implants at thresholds of $50,000 to $100,000 per quality-adjusted life year. Nevertheless, there is currently no evidence to support a definitive clinical recommendation regarding a cost-efficient preferred reconstructive surgical approach [17,18].

Comparative oncological safety and surveillance 

The practice of immediate and post-mastectomy reconstruction has evolved over the years and is now more than a purely restorative effort for the patient; it is very much part of a multidisciplinary approach to breast cancer treatment. The same emphasis has also been placed on oncological safety, patient-reported outcomes, and the integration of the reconstructive process into the overall breast cancer treatment plan by the health care system.

The national survey of Japan conducted by Shien et al. clearly demonstrates that immediate breast reconstruction (IBR) is currently being routinely performed and is feasible. However, there are still several constraints that hinder the complete adoption of this procedure across the entire nation, including institutional variation, limited access to plastic surgeons, and persistent misconceptions regarding the oncological risk associated with IBR for patients who will likely require RT [19]. Compared with the Western world, where IBR is performed at much higher rates, the delay between surgery and IBR authorization is typically much shorter. This highlights the significant role that the structure and cultural context of a country’s health care system play in influencing the timing and uptake of surgical procedures.

Despite historical limitations on performing IBR for patients with advanced disease, emerging literature supports the use of IBR in these patients and challenges previously held beliefs that IBR contributes to increased complication rates or prolongs overall treatment. The systematic review performed by Mattia et al. has clearly demonstrated that breast reconstruction, when performed in de novo metastatic breast cancer patients, does not lead to increased complication rates or delayed initiation of oncological treatment, nor does it improve survival outcomes in appropriately selected patients [20].

This is in contrast to guidelines stating that IBR should not be performed in patients with inflammatory breast cancer. The work by Tadros et al. clearly demonstrates the importance of adhering to the principle of trimodal therapy and the overall detrimental effect of performing early reconstruction with regard to a patient’s overall survival [21].

The increasing use of patient-reported outcomes as a measure of reconstructive success represents a continually growing need, which mirrors the changing nature of what success looks like, no longer being limited to postoperative morbidity. Ramadan et al. further discuss that a lack of cultural and language equivalence impedes the use of well-validated tools for measuring quality of life in low- and middle-income nations, but go further to show that the need for well-validated translations of the BREAST-Q tool exists so that all patients can have a fair measure of success, which further relates to the use of well-validated data for oncological safety [22].

The optimization of the perioperative pathway has been recognized as a major determinant of reconstructive outcomes. Uhlman et al. reported that there is wide variation in the implementation of Enhanced Recovery After Surgery (ERAS) programs in all areas of plastic surgery due to substantial variability and weak methodology in the implementation of ERAS elements, poor adherence to evidence-based components of the ERAS protocol, and weak adherence to elements such as warming, fluid management, and post-discharge monitoring [23]. These gaps in methodology help explain the variability seen in complication rates in the literature when compared with reconstructive outcomes and underscore the need for a standardized reporting structure to ensure reproducibility of safe and effective ERAS programs for breast reconstruction.

The technical refinement of safety parameters in reconstructive surgery through technology has resulted in improved wound healing and implant placement. Abul et al. demonstrate that the use of closed-incision negative pressure wound therapy (ciNPWT) substantially decreased the incidence and severity of donor-site dehiscence after abdominal-based autologous reconstruction, and that the use of ciNPWT resulted in increased retention of body weight by the recipient after breast reconstruction [24].

Similarly, the research conducted by Størling et al. demonstrates that there are no differences in either the rate of complications or the overall number of complications with implant placement above or below the muscle when modern adjuncts are used. This provides further validation that prepectoral reconstruction is a safe option for appropriately selected non-irradiated patients [25]. These results also represent a shift toward a more rational approach to the selection of reconstructive techniques based on patients’ individual risk profiles, rather than a “technique to avoid” mentality.

Thus, modern breast reconstruction surgery is characterized by the intersection of several factors: oncologic safety, patient-reported outcomes, improved perioperative care, and technical advancements. There is evidence in the literature to support that oncologic safety can be maintained when reconstructive surgery is conducted in accordance with tumor biology principles and appropriate reconstructive sequencing, in conjunction with collaborative system-level approaches that aim to reduce disparities in access to care. Looking ahead, achieving equitable access to high-quality breast reconstruction will depend on synthesizing current treatment guidelines, harmonizing outcome measurement, and incorporating cultural sensitivity when identifying appropriate reconstructive strategies on an individual-case basis [19-25].

Perioperative outcomes, complications, and risk management

Perioperative and early postoperative outcomes in implant-based reconstruction are evaluated through postoperative complications requiring procedural intervention, leading to correct classification into overall complications, major complications, and reconstructive failure. Complications and events include hematoma, infection, wound dehiscence, skin flap necrosis, seroma, capsular contracture, implant malposition or failure, and explantation [26].

Seroma is defined as a localized serous fluid collection that requires aspiration. Surgical site infection is defined according to the Centers for Disease Control and Prevention criteria for deep incisional infection. Skin flap necrosis is characterized by soft-tissue ischemia with eschar formation. Explantation refers to implant removal without immediate replacement, and capsular contracture is identified by implant distortion with an associated aesthetic or functional defect [27].

Major perioperative complications most commonly include severe infections with systemic signs or purulence requiring operative irrigation or debridement, often leading to device exchange or explantation [28]. Predictors of reconstructive failure include severe infection requiring operative intervention, capsular contracture, and complications associated with PMRT [29,30].

The success of immediate implant-based reconstructions depends on correct management of perioperative complications, which must preserve both oncologic radicality and soft tissue recovery limits. Medical practitioners today choose prepectoral breast reconstruction (PBR) over subpectoral breast reconstruction (SBR) based on its ability to eliminate animation deformity and its capacity to reduce postoperative patient discomfort. Research studies employing meta-analytical methods demonstrate that PBR patients develop seromas at higher frequencies; therefore, they require proper drain care and clear counseling regarding expected fluid production [1,31].

Apart from this, complication rates, including surgical site infections and hematomas, do not differ significantly between planes. The thickness of skin flaps between 0.5 and 1 cm is a vital measurement because it determines both blood flow to the dermal tissue and the risk of tissue necrosis due to inadequate perfusion [2,32]. Results from clinical audits show that patients experience infections as early complications in 16.33% of cases, even when using the best available treatment methods [31]. The studied population shows that 10.34% of patients experience delayed initiation of adjuvant therapy due to early complications [31].

Long-term success of the procedure is influenced by the administration of PMRT, which remains the main cause of late reconstruction morbidity [8]. RT is strongly related to a higher incidence of Baker grade III/IV capsular contracture compared to patients who did not receive radiation, and it leads to reconstructive failure in 25.4% of cases [27].

The risk becomes more severe because of biological factors, which include regional nodal involvement. The following factors are associated with the study: BMI values above 30 kg/m² and smoking status. Active smokers develop independent RFs that lead to flap necrosis and implant failure [27].

Risk management needs to address aesthetic outcomes because PBR does not cause muscle-related complications; however, it increases the chance of rippling, which may require additional surgical procedures to achieve better soft-tissue coverage and patient contentment [26].

Patient-centered outcomes and shared decision-making

Recent oncologic and plastic surgery literature increasingly recognizes that the optimal reconstructive strategy following mastectomy cannot be determined solely by technical feasibility or surgeon-assessed success. Instead, reconstruction planning must integrate oncologic safety, patient-reported outcomes, and individual patient values, preferences, and expectations. This approach is further shaped by multidisciplinary collaboration and system-level efforts aimed at ensuring equitable access to high-quality reconstructive care [19,32].

Patient-centered outcomes extend beyond traditional surgical endpoints. They include health-related quality of life, physical well-being, psychosocial functioning, sexual well-being, body image, and satisfaction with breast appearance. Collectively, these domains are now regarded as core components of outcome assessment in modern breast reconstruction [19,32].

Validated patient-reported outcome measures (PROMs), particularly the BREAST-Q reconstruction module, have become central instruments for capturing patient perspectives. Evidence from systematic reviews and large observational cohorts indicates that autologous tissue-based reconstruction is associated with higher long-term satisfaction and more favorable psychosocial and sexual well-being compared with implant-based reconstruction. These differences tend to become more apparent with longer follow-up [32].

In patients undergoing immediate reconstruction after neoadjuvant therapy, contemporary comparative data demonstrate that autologous reconstruction is associated with significantly higher patient satisfaction scores than implant-based reconstruction (72.22 ± 18.26 vs. 65.52 ± 19.50, p = 0.035). During the tissue-expander phase of implant-based reconstruction, significantly fewer patients report good-to-excellent aesthetic outcomes compared with autologous reconstruction (65.6% vs. 89.5%, p = 0.002). These findings illustrate the dynamic nature of patient experience across reconstructive stages [33].

Importantly, these differences reflect modality-specific trade-offs rather than universal superiority. Autologous reconstruction is more frequently associated with softer tissue characteristics and improved long-term symmetry. Implant-based reconstruction, by contrast, offers shorter operative time and faster initial recovery but carries higher risks of infection, reconstruction failure, and lower patient satisfaction, particularly in the setting of neoadjuvant therapy and RT [32,33].

Taken together, these observations highlight the need to move beyond a purely technique-centered paradigm toward an explicitly patient-centered reconstructive framework. Shared decision-making represents a critical mechanism for translating this evidence into individualized care. This process entails providing patients with balanced, evidence-based information regarding risks, benefits, and long-term implications of each reconstructive option, while actively eliciting patient goals, cultural context, and personal priorities. Current guidelines emphasize that harmonizing outcome measurement, applying PROM data, and incorporating patient preferences are fundamental to selecting appropriate reconstructive strategies on a case-by-case basis [32,33].

Collectively, these data support a paradigm in which breast reconstruction planning is conceptualized not as a purely technical exercise but as a value-sensitive clinical decision grounded in robust patient-centered outcomes and operationalized through structured shared decision-making [19,32,33].

## Conclusions

The post-mastectomy breast reconstruction panorama now operates under a value-based system, which delivers patient-centered care. This study highlights that implant-based reconstruction offers a less invasive surgical profile, avoids animation deformity, but remains highly sensitive to the adverse effects of post-mastectomy radiotherapy, including an increased risk of capsular contracture and reconstructive failure. In contrast, autologous reconstruction continues to provide the best solution for long-term patient satisfaction. This technique offers better psychosocial and sexual well-being outcomes.

The two methods prove to be oncologically safe and do not interfere with adjuvant therapy timelines in a multidisciplinary setting, according to current evidence. The process of selecting reconstructive methods requires a structured decision-making approach. This process must evaluate the patient’s biological risk factors against their long-term aesthetic goals and oncological treatment plan. To guarantee equitable and optimal care, future initiatives should concentrate on standardizing ERAS protocols and increasing the use of validated patient-reported outcomes worldwide.
